# A three-part model for the self-controlled case series design to estimate and characterize adverse event risk in an overlapping risk period after multiple vaccines: application to ischemic stroke following Pfizer-BioNTech bivalent COVID-19 vaccine and influenza vaccine

**DOI:** 10.1093/aje/kwaf115

**Published:** 2025-06-04

**Authors:** Stanley Xu, Lina S Sy, Xuan Huang, Vennis Hong, Bing Han, Katia J Bruxvoort, Bruno Lewin, Kimberly J Holmquist, Lei Qian

**Affiliations:** Department of Research and Evaluation, Kaiser Permanente Southern California, Pasadena, CA, United States; Department of Health Systems Science, Kaiser Permanente Bernard J. Tyson School of Medicine, Pasadena, CA, United States; Department of Research and Evaluation, Kaiser Permanente Southern California, Pasadena, CA, United States; Department of Research and Evaluation, Kaiser Permanente Southern California, Pasadena, CA, United States; Department of Research and Evaluation, Kaiser Permanente Southern California, Pasadena, CA, United States; Department of Research and Evaluation, Kaiser Permanente Southern California, Pasadena, CA, United States; Department of Research and Evaluation, Kaiser Permanente Southern California, Pasadena, CA, United States; Department of Epidemiology, School of Public Health, University of Alabama at Birmingham, Birmingham, AL, United States; Department of Research and Evaluation, Kaiser Permanente Southern California, Pasadena, CA, United States; Department of Clinical Science, Kaiser Permanente Bernard J. Tyson School of Medicine, Pasadena, CA, United States; Department of Research and Evaluation, Kaiser Permanente Southern California, Pasadena, CA, United States; Department of Research and Evaluation, Kaiser Permanente Southern California, Pasadena, CA, United States

**Keywords:** self-controlled case series, coadministration, overlapping risk period, simulation, vaccine safety study, ischemic stroke, mRNA COVID-19 vaccines, influenza vaccines

## Abstract

This study proposes a three-part model to assess and characterize the risk of serious adverse events when 2 vaccines are administered on the same day or in close proximity within a self-controlled case series framework. Simulations showed that the three-part model yielded unbiased relative incidences (RIs) after each vaccination and during the overlapping risk period, while censoring follow-up at dose 2 reduced estimation precision but produced unbiased point estimates. Assuming positive multiplicative and positive additive effects, including the overlapping risk period in the first risk interval overestimated the RI after the first dose by 6.0%-26.0%, while including it in the second overestimated the second RI by 7.3%-34.0%. Overall analysis using the three-part model found no increased ischemic stroke risk 42 days after Pfizer-BioNTech bivalent COVID-19 vaccination or after influenza vaccination or during the overlapping risk period among Kaiser Permanente Southern California members <65 years. Among those with a prior-year history of SARS-CoV-2 infection, the overlapping period showed a significantly increased risk (RI = 2.74 [95% CI, 1.07-7.07]), indicating both positive multiplicative and additive effects. Further research is needed to validate these ischemic stroke findings with chart review confirmation and to apply the model to other vaccination scenarios.

## Introduction

Past vaccine safety studies have shown that the risk intervals for some serious adverse events (SAEs) can last weeks after vaccination. For example, in studies examining the association between measles-mumps-rubella (MMR) vaccination and immune thrombocytopenic purpura (ITP) among children aged 12-23 months, it was found that the risk of ITP was elevated 3.2-14.6-fold 1-42 days after MMR vaccination.^1^^,^^2^ In another study, an increased risk of Guillain–Barré syndrome (GBS) was observed 1-42 days following administration of monovalent inactivated vaccine against H1N1.^3^ Because of the weeks-long risk interval, an overlapping risk period can occur when different vaccines are administered on the same day or in close proximity. Coadministration of different vaccines on the same day, such as COVID-19 and influenza vaccines, is a strategy recommended by the Centers for Disease Control and Prevention (CDC) to enhance vaccination coverage and prevent missed opportunities.^4^ Coadministration has been shown to improve vaccination rates by reducing the number of healthcare visits required, which is especially beneficial during pandemics or peak influenza seasons.^5^ Studies have demonstrated that administering COVID-19 and influenza vaccines simultaneously is generally safe and effective. For instance, a randomized controlled trial showed that coadministration of COVID-19 and influenza vaccines did not compromise the immune response to either vaccine, while maintaining a favorable safety profile.^6^ Another study reported similar immunogenicity and no significant increase in adverse events when COVID-19 and influenza vaccines were given together compared to when they were given as separate administrations.^7^ However, several studies have shown inconsistent results regarding the potential association between ischemic stroke and bivalent COVID-19 vaccination coadministered with influenza vaccination on the same day. Specifically, a self-controlled case series (SCCS) study conducted in England reported no increased risk of ischemic stroke following coadministration of bivalent COVID-19 and influenza vaccines among individuals aged 65 years and older.^8^ Similarly, a study among Medicare beneficiaries aged 65 and older showed no significantly elevated risk for stroke immediately after receiving bivalent COVID-19 vaccine.^9^ However, among Medicare beneficiaries who received a bivalent COVID-19 vaccine along with a high-dose or adjuvanted influenza vaccine, there was a significant association with nonhemorrhagic stroke within 22 to 42 days post-vaccination for the Pfizer-BioNTech bivalent COVID-19 vaccine and a significant association with transient ischemic attack within 1 to 21 days post-vaccination for the Moderna bivalent COVID-19 vaccine.^9^ Another SCCS study identified a marginally statistically significant elevated risk of ischemic stroke among individuals <65 years who received the Pfizer-BioNTech bivalent COVID-19 vaccine and the influenza vaccine on the same day.^10^

Overlapping risk periods can also occur when 2 doses of the same vaccine are administered in close succession, such as in the 2-dose primary series of mRNA COVID-19 vaccines (Pfizer-BioNTech COVID-19 vaccine [BNT162b2] and Moderna COVID-19 vaccine [mRNA-1273]). If 2 doses of the same vaccine are administered in close proximity, there could be an overlapping risk period where the 2 risk intervals overlap. It is methodologically challenging and clinically important to appropriately assess and characterize the risk of SAEs for 2 different vaccines administered together (or in close succession) or the 2-dose primary series of mRNA COVID-19 vaccines due to the overlapping risk intervals.

Past studies considered only the risk of SAEs following bivalent COVID-19 vaccination alone and following coadministration with the influenza vaccine on the same day, without accounting for the risk after influenza vaccination alone and coadministration of bivalent COVID-19 vaccine in close proximity but not on the same day. In this study, we propose a three-part model within the SCCS framework to estimate the risk associated with each of the 2 vaccines individually, as well as during their overlapping risk period, and to further characterize the risk during this overlap. In a simulation study, we compared the three-part model to three existing methods that avoid the overlapping risk period issue.^11^^,^^12^ We applied the three-part model to assess the risk of ischemic stroke after Pfizer-BioNTech bivalent COVID-19 vaccination and influenza vaccination among Kaiser Permanente Southern California (KPSC) members <65 years during the 2022-2023 season.

## Methods

### Standard SCCS

The standard SCCS design was originally developed for studying transient exposures like vaccinations and acute recurrent outcomes such as febrile convulsions and aseptic meningitis.^12^^,^^13^ In this design, an individual’s observation period is divided into risk and control intervals, allowing for the comparison of incidence rates within the same individual and implicit adjustment for time-invariant confounders. Self-controlled case series data are typically analyzed using conditional Poisson regression or fixed effect models, conditioning on the total number of SAEs within each individual.^12-14^ Standard SCCS can be applied for multiple exposures. When multiple doses are administered on the same day or different days, an interaction term for the multiple exposures is included in the SCCS model to estimate the risk during the overlapping risk period^12^; however, the parameterization for the overlapping risk period is a function of the risk after each vaccine and does not fully characterize the risk during the overlapping risk period.

### A three-part model for two doses of vaccines

**Figure 1:**
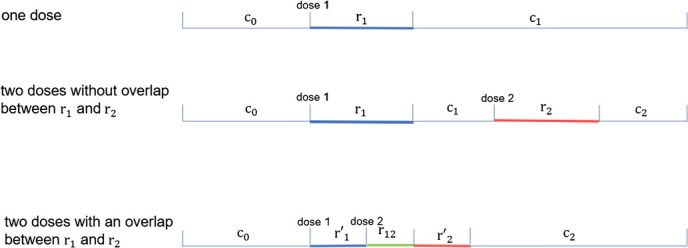
Risk and control intervals for (A) an individual who received 1 dose, (B) an individual who received 2 doses without overlap between ${\mathrm{r}}_1$ and ${\mathrm{r}}_2$, and (C) an individual who received 2 doses with an overlap (${\mathrm{r}}_{12}$) between ${\mathrm{r}}_1$ and ${\mathrm{r}}_2$.

In this study, we considered risk of SAEs after coadministration of multiple doses of vaccines both on the same day and in close proximity in addition to risk of SAEs after each dose. Let ${\mathrm{d}}_1$ and ${\mathrm{d}}_2$ represent the 2 doses, and ${\mathrm{r}}_1$ and ${\mathrm{r}}_2$ represent the 2 risk intervals after ${\mathrm{d}}_1$ and ${\mathrm{d}}_2$, respectively. The control intervals, risk intervals, and a possible overlapping risk period are illustrated in Figure 1.

These doses can be the 2 doses of the same vaccine, such as the 2-dose primary series of mRNA COVID-19 vaccines, or 1 dose each of 2 different vaccines, such as the bivalent COVID-19 vaccine and the influenza vaccine. Further, let ${\mathrm{\beta}}_1$ and ${\mathrm{\beta}}_2$ represent the coefficients for the relative incidence (RI) of a SAE of interest during ${\mathrm{r}}_1$ and ${\mathrm{r}}_2$, respectively, as compared to control intervals. For those who received 2 doses, if ${\mathrm{d}}_2$ was administered after ${\mathrm{r}}_1$ (there was no overlap between ${\mathrm{r}}_1$ and ${\mathrm{r}}_2$), assuming known risk intervals and a constant risk, and conditioning on the total number of SAEs within an individual, the conditional likelihood can be calculated as follows:


(1) \begin{align*}{L}_{NO}=&{\left[\frac{C_0\exp \left({\mathbf{X}}_{{\mathrm{c}}_0}\boldsymbol{\mathrm{\theta}} \right)}{\begin{matrix}C_0\exp \left({\mathbf{X}}_{{\mathrm{c}}_0}\boldsymbol{\mathrm{\theta}} \right)+{R}_1\exp \left({\mathbf{X}}_{{\mathrm{r}}_1}\boldsymbol{\mathrm{\theta}} +{\mathrm{\beta}}_1\right)\\+{C}_1\exp \left({\mathbf{X}}_{{\mathrm{c}}_1}\boldsymbol{\mathrm{\theta}} \right)+{R}_2\exp \left({\mathbf{X}}_{{\mathrm{r}}_2}\boldsymbol{\mathrm{\theta}} +{\mathrm{\beta}}_2\right)+{C}_2\exp \left({\mathbf{X}}_{{\mathrm{c}}_2}\boldsymbol{\mathrm{\theta}} \right)\end{matrix}}\right]}^{y_{c_0}} \notag \\ &{\left[\frac{ {R}_1\exp \left({\mathbf{X}}_{{\mathrm{r}}_1}\boldsymbol{\mathrm{\theta}} +{\mathrm{\beta}}_1\right)}{\begin{matrix} C_0\exp \left({\mathbf{X}}_{{\mathrm{c}}_0}\boldsymbol{\mathrm{\theta}} \right)+{R}_1\exp \left({\mathbf{X}}_{{\mathrm{r}}_1}\boldsymbol{\mathrm{\theta}} +{\mathrm{\beta}}_1\right)+{C}_1\exp \left({\mathbf{X}}_{{\mathrm{c}}_1}\boldsymbol{\mathrm{\theta}} \right)\\+{R}_2\exp \left({\mathbf{X}}_{{\mathrm{r}}_2}\boldsymbol{\mathrm{\theta}} +{\mathrm{\beta}}_2\right)+{C}_2\exp \left({\mathbf{X}}_{{\mathrm{c}}_2}\boldsymbol{\mathrm{\theta}} \right)\end{matrix}}\right]}^{y_{r_1}} \notag \\ &{\left[\frac{C_1\exp \left({\mathbf{X}}_{{\mathrm{c}}_1}\boldsymbol{\mathrm{\theta}} \right)}{\begin{matrix} C_0\exp \left({\mathbf{X}}_{{\mathrm{c}}_0}\boldsymbol{\mathrm{\theta}} \right)+{R}_1\exp \left({\mathbf{X}}_{{\mathrm{r}}_1}\boldsymbol{\mathrm{\theta}} +{\mathrm{\beta}}_1\right)+{C}_1\exp \left({\mathbf{X}}_{{\mathrm{c}}_1}\boldsymbol{\mathrm{\theta}} \right)\\+{R}_2\exp \left({\mathbf{X}}_{{\mathrm{r}}_2}\boldsymbol{\mathrm{\theta}} +{\mathrm{\beta}}_2\right)+{C}_2\exp \left({\mathbf{X}}_{{\mathrm{c}}_2}\boldsymbol{\mathrm{\theta}} \right)\end{matrix}}\right]}^{y_{c_1}}\notag \\ &{\left[\frac{R_2\exp \left({\mathbf{X}}_{{\mathrm{r}}_2}\boldsymbol{\mathrm{\theta}} +{\mathrm{\beta}}_2\right)}{\begin{matrix}C_0\exp \left({\mathbf{X}}_{{\mathrm{c}}_0}\boldsymbol{\mathrm{\theta}} \right)+{R}_1\exp \left({\mathbf{X}}_{{\mathrm{r}}_1}\boldsymbol{\mathrm{\theta}} +{\mathrm{\beta}}_1\right)+{C}_1\exp \left({\mathbf{X}}_{{\mathrm{c}}_1}\boldsymbol{\mathrm{\theta}} \right)\\+{R}_2\exp \left({\mathbf{X}}_{{\mathrm{r}}_2}\boldsymbol{\mathrm{\theta}} +{\mathrm{\beta}}_2\right)+{C}_2\exp \left({\mathbf{X}}_{{\mathrm{c}}_2}\boldsymbol{\mathrm{\theta}} \right)\end{matrix}}\right]}^{y_{r_2}} \notag \\ &{\left[\frac{C_2\exp \left({\mathbf{X}}_{{\mathrm{c}}_2}\boldsymbol{\mathrm{\theta}} \right)}{\begin{matrix}C_0\exp \left({\mathbf{X}}_{{\mathrm{c}}_0}\boldsymbol{\mathrm{\theta}} \right)+{R}_1\exp \left({\mathbf{X}}_{{\mathrm{r}}_1}\boldsymbol{\mathrm{\theta}} +{\mathrm{\beta}}_1\right)+{C}_1\exp \left({\mathbf{X}}_{{\mathrm{c}}_1}\boldsymbol{\mathrm{\theta}} \right)\\+{R}_2\exp \left({\mathbf{X}}_{{\mathrm{r}}_2}\boldsymbol{\mathrm{\theta}} +{\mathrm{\beta}}_2\right)+{C}_2\exp \left({\mathbf{X}}_{{\mathrm{c}}_2}\boldsymbol{\mathrm{\theta}} \right)\end{matrix}}\right]}^{y_{c_2}} \end{align*}

where ${C}_0$, ${R}_1$, ${C}_1$, ${R}_2$, and ${C}_2$ represent person-time in days for the partitioned intervals (Figure 1); ${y}_{c_0}$, ${y}_{r_1},{y}_{c_1},{y}_{r_2},\mathrm{and}\ {y}_{c_2}$ are numbers of SAEs in these intervals (for rare SAE, they are binary values of 1 or 0); ${\mathrm{\beta}}_1$ and ${\mathrm{\beta}}_2$ are the coefficients for the vaccination effects after doses 1 and 2; ${\mathbf{X}}_{c_0}$, ${\mathbf{X}}_{{\mathrm{r}}_1},{\mathbf{X}}_{{\mathrm{c}}_1},{\mathbf{X}}_{{\mathrm{r}}_2},\mathrm{and}\ {\mathbf{X}}_{{\mathrm{c}}_2}$ represent the row vector of time-varying covariates for these intervals; and $\boldsymbol{\mathrm{\theta}}$ represents the column vector of corresponding coefficients. Time-varying covariates include, but are not limited to, factors such as seasonality. Although seasonality and other time-varying covariates were incorporated into the theoretical models, for simplicity, they were not included in the simulation.

If ${\mathrm{d}}_2$ was administered during ${\mathrm{r}}_1$, there was an overlap between ${\mathrm{r}}_1$ and ${\mathrm{r}}_2$. Let ${\mathrm{r}}_{12}$ denote the overlapping risk period with a length of ${R}_{12}$ and ${\mathrm{\beta}}_{12}$ denote the coefficient for the ${\mathrm{RI}}_{12}$ of an SAE. The conditional likelihood for individuals with an overlapping risk period can be calculated as: 


(2) \begin{align*}{L}_{OL}=&{\left[\frac{C_0\exp \left({\mathbf{X}}_{{\mathrm{c}}_0}\boldsymbol{\mathrm{\theta}} \right)}{\begin{matrix}C_0\exp \left({\mathbf{X}}_{{\mathrm{c}}_0}\boldsymbol{\mathrm{\theta}} \right)+{R}_1^{\prime}\exp \left({\mathbf{X}}_{{\mathrm{r}}_1^{\prime }}\boldsymbol{\mathrm{\theta}} +{\mathrm{\beta}}_1\right)+{R}_{12}\exp \left({\mathbf{X}}_{{\mathrm{r}}_{12}}\boldsymbol{\mathrm{\theta}} +{\mathrm{\beta}}_{12}\right)\\+{R}_2^{\prime}\exp \left({\mathbf{X}}_{{\mathrm{r}}_2^{\prime }}\boldsymbol{\mathrm{\theta}} +{\mathrm{\beta}}_2\right)+{C}_2\exp \left({\mathbf{X}}_{{\mathrm{c}}_2}\boldsymbol{\mathrm{\theta}} \right)\end{matrix}}\right]}^{y_{c_0}} \notag \\ &{\left[\frac{R_1^{\prime}\exp \left({\mathbf{X}}_{{\mathrm{r}}_1^{\prime }}\boldsymbol{\mathrm{\theta}} +{\mathrm{\beta}}_1\right)}{\begin{matrix} C_0\exp \left({\mathbf{X}}_{{\mathrm{c}}_0}\boldsymbol{\mathrm{\theta}} \right)+{R}_1^{\prime}\exp \left({\mathbf{X}}_{{\mathrm{r}}_1^{\prime }}\boldsymbol{\mathrm{\theta}} +{\mathrm{\beta}}_1\right)+{R}_{12}\exp \left({\mathbf{X}}_{{\mathrm{r}}_{12}}\boldsymbol{\mathrm{\theta}} +{\mathrm{\beta}}_{12}\right)\\+{R}_2^{\prime}\exp \left({\mathbf{X}}_{{\mathrm{r}}_2^{\prime }}\boldsymbol{\mathrm{\theta}} +{\mathrm{\beta}}_2\right)+{C}_2\exp \left({\mathbf{X}}_{{\mathrm{c}}_2}\boldsymbol{\mathrm{\theta}} \right)\end{matrix}}\right]}^{y_{r_1^{\prime }}} \notag \\ &{\left[\frac{ {R}_{12}\exp \left({\mathbf{X}}_{{\mathrm{r}}_{12}}\boldsymbol{\mathrm{\theta}} +{\mathrm{\beta}}_{12}\right)}{\begin{matrix} C_0\exp \left({\mathbf{X}}_{{\mathrm{c}}_0}\boldsymbol{\mathrm{\theta}} \right)+{R}_1^{\prime}\exp \left({\mathbf{X}}_{{\mathrm{r}}_1^{\prime }}\boldsymbol{\mathrm{\theta}} +{\mathrm{\beta}}_1\right)+{R}_{12}\exp \left({\mathbf{X}}_{{\mathrm{r}}_{12}}\boldsymbol{\mathrm{\theta}} +{\mathrm{\beta}}_{12}\right)\\+{R}_2^{\prime}\exp \left({\mathbf{X}}_{{\mathrm{r}}_2^{\prime }}\boldsymbol{\mathrm{\theta}} +{\mathrm{\beta}}_2\right)+{C}_2\exp \left({\mathbf{X}}_{{\mathrm{c}}_2}\boldsymbol{\mathrm{\theta}} \right)\end{matrix}}\right]}^{y_{r_{12}}}\notag \\ &{\left[\frac{R_2^{\prime}\exp \left({\mathbf{X}}_{{\mathrm{r}}_2^{\prime }}\boldsymbol{\mathrm{\theta}} +{\mathrm{\beta}}_1\right)}{\begin{matrix}C_0\exp \left({\mathbf{X}}_{{\mathrm{c}}_0}\boldsymbol{\mathrm{\theta}} \right)+{R}_1^{\prime}\exp \left({\mathbf{X}}_{{\mathrm{r}}_1^{\prime }}\boldsymbol{\mathrm{\theta}} +{\mathrm{\beta}}_1\right)+{R}_{12}\exp \left({\mathbf{X}}_{{\mathrm{r}}_{12}}\boldsymbol{\mathrm{\theta}} +{\mathrm{\beta}}_{12}\right)\\+{R}_2^{\prime}\exp \left({\mathbf{X}}_{{\mathrm{r}}_2^{\prime }}\boldsymbol{\mathrm{\theta}} +{\mathrm{\beta}}_2\right)+{C}_2\exp \left({\mathbf{X}}_{{\mathrm{c}}_2}\boldsymbol{\mathrm{\theta}} \right)\end{matrix}}\right]}^{y_{r_2^{\prime }}} \notag \\ &{\left[\frac{C_2\exp \left({\mathbf{X}}_{{\mathrm{c}}_2}\boldsymbol{\mathrm{\theta}} \right)}{\begin{matrix}C_0\exp \left({\mathbf{X}}_{{\mathrm{c}}_0}\boldsymbol{\mathrm{\theta}} \right)+{R}_1^{\prime}\exp \left({\mathbf{X}}_{{\mathrm{r}}_1^{\prime }}\boldsymbol{\mathrm{\theta}} +{\mathrm{\beta}}_1\right)+{R}_{12}\exp \left({\mathbf{X}}_{{\mathrm{r}}_{12}}\boldsymbol{\mathrm{\theta}} +{\mathrm{\beta}}_{12}\right)\\+{R}_2^{\prime}\exp \left({\mathbf{X}}_{{\mathrm{r}}_2^{\prime }}\boldsymbol{\mathrm{\theta}} +{\mathrm{\beta}}_2\right)+{C}_2\exp \left({\mathbf{X}}_{{\mathrm{c}}_2}\boldsymbol{\mathrm{\theta}} \right)\end{matrix}}\right]}^{y_{c_2}} \end{align*}

where ${R}_1^{\prime }={R}_1-{R}_{12}$ and ${R}_2^{\prime }={R}_2-{R}_{12}$.

For those who received only 1 dose, the likelihood function is:


(3) \begin{align*} {L}_{d1}=&{\left[\frac{C_0\exp \left({\mathbf{X}}_{c_0}\boldsymbol{\mathrm{\theta}} \right)}{C_0\exp \left({\mathbf{X}}_{{\mathrm{c}}_0}\boldsymbol{\mathrm{\theta}} \right)+{R}_1\exp \left({\mathbf{X}}_{{\mathrm{r}}_1}\boldsymbol{\mathrm{\theta}} +{\mathrm{\beta}}_1\right)+{C}_1\exp \left({\mathbf{X}}_{{\mathrm{c}}_1}\boldsymbol{\mathrm{\theta}} \right)}\right]}^{y_{c_0}} \notag \\ &{\left[\frac{\ {R}_1\exp \left({\mathbf{X}}_{r_1}\boldsymbol{\mathrm{\theta}} +{\mathrm{\beta}}_1\right)}{C_0\exp \left({\mathbf{X}}_{{\mathrm{c}}_0}\boldsymbol{\mathrm{\theta}} \right)+{R}_1\exp \left({\mathbf{X}}_{{\mathrm{r}}_1}\boldsymbol{\mathrm{\theta}} +{\mathrm{\beta}}_1\right)+{C}_1\exp \left({\mathbf{X}}_{{\mathrm{c}}_1}\boldsymbol{\mathrm{\theta}} \right)}\right]}^{y_{r_1}} \notag \\ &{\left[\frac{C_1\exp \left({\boldsymbol{X}}_{c_1}\boldsymbol{\mathrm{\theta}} \right)}{C_0\exp \left({\mathbf{X}}_{{\mathrm{c}}_0}\boldsymbol{\mathrm{\theta}} \right)+{R}_1\exp \left({\mathbf{X}}_{{\mathrm{r}}_1}\boldsymbol{\mathrm{\theta}} +{\mathrm{\beta}}_1\right)+{C}_1\exp \left({\mathbf{X}}_{{\mathrm{c}}_1}\boldsymbol{\mathrm{\theta}} \right)}\right]}^{y_{c_1}}. \end{align*}

Maximum likelihood estimates of ${\mathrm{\beta}}_1$, ${\mathrm{\beta}}_2$, and ${\mathrm{\beta}}_{12}$ can be obtained by maximizing the likelihood functions. Estimated RIs after doses 1 and 2 and during the overlapping risk period are ${\hat{\mathrm{RI}}}_1={\mathrm{e}}^{{\hat{\mathrm{\beta}}}_1},$  ${\hat{\mathrm{RI}}}_2={\mathrm{e}}^{{\hat{\mathrm{\beta}}}_2}$, and ${\hat{\mathrm{RI}}}_{12}={\mathrm{e}}^{{\hat{\mathrm{\beta}}}_{12}}$.

### Three other methods and their likelihood functions

**Figure 2:**
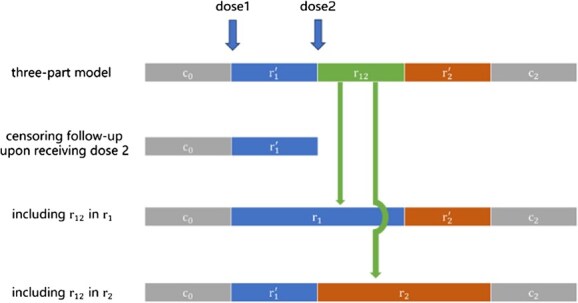
Four methods for handling the overlapping risk period: (A) the three-part model, (B) censoring follow-up upon receiving dose 2, (C) including the overlapping risk period in ${\mathrm{r}}_1$, and (D) including the overlapping risk period in ${\mathrm{r}}_2$.

In addition to the three-part model, 3 other methods have been used to avoid the overlapping risk period issue: (1) censoring follow-up upon receiving dose 2; (2) including the overlapping risk period in ${\mathrm{r}}_1$, although this approach is uncommon; and (3) including the overlapping risk period in ${\mathrm{r}}_2$ (Figure 2).

The likelihood function for the overlapping risk period ${L}_{OL}$ can be modified to model these three other methods.

(1) For censoring follow-up upon receiving dose 2:


(4) \begin{align*} {L}_{OL(1)}=&{\left[\frac{C_0\exp \left({\mathbf{X}}_{{\mathrm{c}}_0}\boldsymbol{\mathrm{\theta}} \right)}{C_0\exp \left({\mathbf{X}}_{{\mathrm{c}}_0}\boldsymbol{\mathrm{\theta}} \right)+{R}_1^{\prime}\exp \left({\mathbf{X}}_{{\mathrm{r}}_1^{\prime }}\boldsymbol{\mathrm{\theta}} +{\mathrm{\beta}}_1\right)}\right]}^{y_{c_0}}\notag \\ &{\left[\frac{R_1^{\prime}\exp \left({\mathbf{X}}_{{\mathrm{r}}_1^{\prime }}\boldsymbol{\mathrm{\theta}} +{\mathrm{\beta}}_1\right)}{C_0\exp \left({\mathbf{X}}_{{\mathrm{c}}_0}\boldsymbol{\mathrm{\theta}} \right)+{R}_1^{\prime}\exp \left({\mathbf{X}}_{{\mathrm{r}}_1^{\prime }}\boldsymbol{\mathrm{\theta}} +{\mathrm{\beta}}_1\right)}\right]}^{y_{r_1^{\prime }}} \end{align*}

where the length of the first risk interval became ${R}_1^{\prime }={R}_1-{R}_{12}$, and the corresponding coefficient remained the same, ${\mathrm{\beta}}_1$.

(2) For including the overlapping risk period in ${\mathrm{r}}_1$:


(5) \begin{align*}{L}_{OL(2)}=&{\left[\frac{C_0\exp \left({\mathbf{X}}_{{\mathrm{c}}_0}\boldsymbol{\mathrm{\theta}} \right)}{\begin{matrix}C_0\exp \left({\mathbf{X}}_{{\mathrm{c}}_0}\boldsymbol{\mathrm{\theta}} \right)+{R}_1\exp \left({\mathbf{X}}_{{\mathrm{r}}_1}\boldsymbol{\mathrm{\theta}} +{\mathrm{\beta}}_1^{\prime}\right)+{R}_2^{\prime}\exp \left({\mathbf{X}}_{{\mathrm{r}}_2^{\prime }}\boldsymbol{\mathrm{\theta}} +{\mathrm{\beta}}_2\right)\\+{C}_2\exp \left({\mathbf{X}}_{{\mathrm{c}}_2}\boldsymbol{\mathrm{\theta}} \right)\end{matrix}}\right]}^{y_{c_0}} \notag \\ &{\left[\frac{{R}_1\exp \left({\mathbf{X}}_{{\mathrm{r}}_1}\boldsymbol{\mathrm{\theta}} +{\mathrm{\beta}}_1^{\prime}\right)}{\begin{matrix} C_0\exp \left({\mathbf{X}}_{{\mathrm{c}}_0}\boldsymbol{\mathrm{\theta}} \right)+{R}_1\exp \left({\mathbf{X}}_{{\mathrm{r}}_1}\boldsymbol{\mathrm{\theta}} +{\mathrm{\beta}}_1^{\prime}\right)+{R}_2^{\prime}\exp \left({\mathbf{X}}_{{\mathrm{r}}_2^{\prime }}\boldsymbol{\mathrm{\theta}} +{\mathrm{\beta}}_2\right)\\+{C}_2\exp \left({\mathbf{X}}_{{\mathrm{c}}_2}\boldsymbol{\mathrm{\theta}} \right)\end{matrix}}\right]}^{y_{r_1}} \notag \\ &{\left[\frac{R_2^{\prime}\exp \left({\mathbf{X}}_{{\mathrm{r}}_2^{\prime }}\boldsymbol{\mathrm{\theta}} +{\mathrm{\beta}}_2\right)}{\begin{matrix} C_0\exp \left({\mathbf{X}}_{{\mathrm{c}}_0}\boldsymbol{\mathrm{\theta}} \right)+{R}_1\exp \left({\mathbf{X}}_{{\mathrm{r}}_1}\boldsymbol{\mathrm{\theta}} +{\mathrm{\beta}}_1^{\prime}\right)+{R}_2^{\prime}\exp \left({\mathbf{X}}_{{\mathrm{r}}_2^{\prime }}\boldsymbol{\mathrm{\theta}} +{\mathrm{\beta}}_2\right)\\+{C}_2\exp \left({\mathbf{X}}_{{\mathrm{c}}_2}\boldsymbol{\mathrm{\theta}} \right)\end{matrix}}\right]}^{y_{r_2^{\prime }}}\notag \\ &{\left[\frac{C_2\exp \left({\mathbf{X}}_{{\mathrm{c}}_2}\boldsymbol{\mathrm{\theta}} \right)}{\begin{matrix}C_0\exp \left({\mathbf{X}}_{{\mathrm{c}}_0}\boldsymbol{\mathrm{\theta}} \right)+{R}_1\exp \left({\mathbf{X}}_{{\mathrm{r}}_1}\boldsymbol{\mathrm{\theta}} +{\mathrm{\beta}}_1^{\prime}\right)+{R}_2^{\prime}\exp \left({\mathbf{X}}_{{\mathrm{r}}_2^{\prime }}\boldsymbol{\mathrm{\theta}} +{\mathrm{\beta}}_2\right)\\+{C}_2\exp \left({\mathbf{X}}_{{\mathrm{c}}_2}\boldsymbol{\mathrm{\theta}} \right)\end{matrix}}\right]}^{y_{c_2}} \end{align*}

where the length of the first risk interval remained the same, but the corresponding coefficient became ${\mathrm{\beta}}_1^{\prime }$ and the length of the second risk interval became ${R}_2^{\prime }={R}_2-{R}_{12}$.

(3) For including the overlapping risk period in ${\mathrm{r}}_2$:


(6) \begin{align*}{L}_{OL(3)}=&{\left[\frac{C_0\exp \left({\mathbf{X}}_{{\mathrm{c}}_0}\boldsymbol{\mathrm{\theta}} \right)}{\begin{matrix}C_0\exp \left({\mathbf{X}}_{{\mathrm{c}}_0}\boldsymbol{\mathrm{\theta}} \right)+{R}_1^{\prime}\exp \left({\mathbf{X}}_{{\mathrm{r}}_1^{\prime }}\boldsymbol{\mathrm{\theta}} +{\mathrm{\beta}}_1\right)+{R}_2\exp \left({\mathbf{X}}_{{\mathrm{r}}_2}\boldsymbol{\mathrm{\theta}} +{\mathrm{\beta}}_2^{\prime}\right)\\+{C}_2\exp \left({\mathbf{X}}_{{\mathrm{c}}_2}\boldsymbol{\mathrm{\theta}} \right)\end{matrix}}\right]}^{y_{c_0}} \notag \\ &{\left[\frac{R_1^{\prime}\exp \left({\mathbf{X}}_{{\mathrm{R}}_1^{\prime }}\boldsymbol{\mathrm{\theta}} +{\mathrm{\beta}}_1\right)}{\begin{matrix} C_0\exp \left({\mathbf{X}}_{{\mathrm{c}}_0}\boldsymbol{\mathrm{\theta}} \right)+{R}_1^{\prime}\exp \left({\mathbf{X}}_{{\mathrm{r}}_1^{\prime }}\boldsymbol{\mathrm{\theta}} +{\mathrm{\beta}}_1\right)+{R}_2\exp \left({\mathbf{X}}_{{\mathrm{r}}_2}\boldsymbol{\mathrm{\theta}} +{\mathrm{\beta}}_2^{\prime}\right)\\+{C}_2\exp \left({\mathbf{X}}_{{\mathrm{c}}_2}\boldsymbol{\mathrm{\theta}} \right)\end{matrix}}\right]}^{y_{r{\prime}_1}} \notag \\ &{\left[\frac{{R}_2\exp \left({\mathbf{X}}_{{\mathrm{r}}_2}\boldsymbol{\mathrm{\theta}} +{\mathrm{\beta}}_2^{\prime}\right)}{\begin{matrix} C_0\exp \left({\mathbf{X}}_{{\mathrm{c}}_0}\boldsymbol{\mathrm{\theta}} \right)+{R}_1^{\prime}\exp \left({\mathbf{X}}_{{\mathrm{r}}_1^{\prime }}\boldsymbol{\mathrm{\theta}} +{\mathrm{\beta}}_1\right)+{R}_2\exp \left({\mathbf{X}}_{{\mathrm{r}}_2}\boldsymbol{\mathrm{\theta}} +{\mathrm{\beta}}_2^{\prime}\right)\\+{C}_2\exp \left({\mathbf{X}}_{{\mathrm{c}}_2}\boldsymbol{\mathrm{\theta}} \right)\end{matrix}}\right]}^{y_{r_2}}\notag \\ &{\left[\frac{C_2\exp \left({\mathbf{X}}_{{\mathrm{c}}_2}\boldsymbol{\mathrm{\theta}} \right)}{\begin{matrix}C_0\exp \left({\mathbf{X}}_{{\mathrm{c}}_0}\boldsymbol{\mathrm{\theta}} \right)+{R}_1^{\prime}\exp \left({\mathbf{X}}_{{\mathrm{r}}_1^{\prime }}\boldsymbol{\mathrm{\theta}} +{\mathrm{\beta}}_1\right)+{R}_2\exp \left({\mathbf{X}}_{{\mathrm{r}}_2}\boldsymbol{\mathrm{\theta}} +{\mathrm{\beta}}_2^{\prime}\right)\\+{C}_2\exp \left({\mathbf{X}}_{{\mathrm{c}}_2}\boldsymbol{\mathrm{\theta}} \right)\end{matrix}}\right]}^{y_{c_2}} \end{align*}

where the length of the second risk interval remained the same, but the corresponding coefficient became ${\mathrm{\beta}}_2^{\prime }$, and the length of the first risk interval became ${R}_1^{\prime }={R}_1-{R}_{12}$.

### Characterization of the risk during the overlapping risk period

Given the nonlinear relationship (log link) between risk of SAEs and vaccination, we used specific definitions (Table 1) to characterize the risk during the overlapping risk period.^15^ Let $\mathrm{A}={\mathrm{RI}}_1+{\mathrm{RI}}_2-1$ denote the threshold function for defining an additive effect. If ${\widehat{\mathrm{RI}}}_{12}=\hat{\mathrm{A}}={\widehat{\mathrm{RI}}}_1+{\widehat{\mathrm{RI}}}_2-1$, there is no interaction on the additive scale for exposure to both vaccines; if ${\widehat{\mathrm{RI}}}_{12}>\hat{\mathrm{A}}$, the risk during the overlapping risk period is positive additive; if ${\widehat{\mathrm{RI}}}_{12}<\hat{\mathrm{A}}$, the risk during the overlapping risk period is negative additive. For the multiplicative effect, let $\mathrm{M}=\left({\mathrm{RI}}_1\right)\left({\mathrm{RI}}_2\right)$ denote the threshold function for defining a multiplicative effect. Similarly, if ${\widehat{\mathrm{RI}}}_{12}=\hat{\mathrm{M}}$, there is no interaction on the multiplicative scale for exposure to both vaccines; if ${\widehat{\mathrm{RI}}}_{12}>\hat{\mathrm{M}}$, the risk is positive multiplicative; if ${\widehat{\mathrm{RI}}}_{12}<\hat{\mathrm{M}}$, the risk is negative multiplicative.

**Table 1:** Threshold functions for a multiplicative effect and an additive effect, and definitions of four effect types.

Threshold function for a multiplicative effect	$\mathrm{M}=\left({\mathrm{RI}}_{\mathbf{1}}\right)\left({\mathrm{RI}}_{\mathbf{2}}\right)$	${\mathrm{e}}^{{{\mathrm{\beta}}}_{\mathbf{1}\mathbf{2}}}={\mathrm{e}}^{{{\mathrm{\beta}}}_{\mathbf{1}}}{\mathrm{e}}^{{{\mathrm{\beta}}}_{\mathbf{2}}}$
A positive multiplicative effect	${\mathrm{RI}}_{12}>\mathrm{M}$	${\mathrm{e}}^{\mathrm{\beta}_{12}}>{\mathrm{e}}^{\mathrm{\beta}_1}{\mathrm{e}}^{\mathrm{\beta}_2}$
A negative multiplicative effect	${\mathrm{RI}}_{12}<\mathrm{M}$	${\mathrm{e}}^{\mathrm{\beta}_{12}}<{\mathrm{e}}^{\mathrm{\beta}_1}{\mathrm{e}}^{\mathrm{\beta}_2}$
Threshold function for an additive effect	$\mathrm{A}={\mathrm{RI}}_1+{\mathrm{RI}}_2-1$	${\mathrm{e}}^{\mathrm{\beta}_{12}}=\left({\mathrm{e}}^{\mathrm{\beta}_1}+{\mathrm{e}}^{\mathrm{\beta}_2}\right)-1$
A positive additive effect	${\mathrm{RI}}_{12}>\mathrm{A}$	${\mathrm{e}}^{\mathrm{\beta}_{12}}>\left({\mathrm{e}}^{\mathrm{\beta}_1}+{\mathrm{e}}^{\mathrm{\beta}_2}\right)-1$
A negative additive effect	${\mathrm{RI}}_{12}<\mathrm{A}$	${\mathrm{e}}^{\mathrm{\beta}_{12}}<\left({\mathrm{e}}^{\mathrm{\beta}_1}+{\mathrm{e}}^{\mathrm{\beta}_2}\right)-1$

To illustrate these effects, we assume ${\mathrm{\beta}}_1={\mathrm{\beta}}_2=\mathrm{\beta}$, then $\mathrm{A}=2{\mathrm{e}}^{\mathrm{\beta}}-1$ and $\mathrm{M}={\mathrm{e}}^{2\mathrm{\beta}}$. Vaccine safety studies often focus on an increased risk of SAE, meaning ${\mathrm{\beta}}_1>0$ and ${\mathrm{\beta}}_2>0$, in the first quadrant of a coordinate plane where $\mathrm{A}$ and $\mathrm{M}$ can be plotted. The first quadrant is divided into three areas: (1) when ${\mathrm{RI}}_{12}>\mathrm{M}$, the risk during ${\mathrm{r}}_{12}$ is both positive multiplicative and positive additive; (2) when $\mathrm{A}<{\mathrm{RI}}_{12}<\mathrm{M}$, the risk during ${\mathrm{r}}_{12}$ is positive additive but negative multiplicative; (3) when ${\mathrm{RI}}_{12}<\mathrm{A}$, the risk during ${\mathrm{r}}_{12}$ is both negative additive and negative multiplicative.

Compared to ${\mathrm{RI}}_1$ and ${\mathrm{RI}}_2$, ${\mathrm{RI}}_{12}>\mathrm{M}$ indicates a dramatic increase in risk, $\mathrm{A}<{\mathrm{RI}}_{12}<\mathrm{M}$ indicates a moderate increase in risk, and ${\mathrm{RI}}_{12}<\mathrm{A}$, indicates no increase in risk during the overlapping risk period compared to the risk after each vaccination.

### Simulations

#### Simulation algorithm and analyses

We conducted extensive simulation studies to (1) evaluate the performance of 3 existing methods that estimate only risks after doses 1 and 2 and the performance of the three-part model that estimates risks after doses 1 and 2 as well as during the overlapping risk period; (2) assess the ability of the three-part model to characterize the risk during the overlapping period. Self-controlled case series data for 2 doses during an observation period were simulated as described in a previous study.^16^ Briefly, in each simulated dataset, 10 000 hypothetical individuals who received dose 1 were assumed to have an observation period of 120 days ($t=1\ \mathrm{to}\ 120)$, consisting of 3 control intervals (${\mathrm{c}}_0,{\mathrm{c}}_1,$ and ${\mathrm{c}}_2$) and 2 risk intervals (${R}_1=28$ days and ${R}_2=28$ days). We assumed all individuals received dose 1 on day 15, with ${C}_0=14$ days. Among the 10 000 individuals, 20% did not receive dose 2, with ${C}_1=78$ days; 30% received dose 2 uniformly between day 44 and day 90, and ${\mathrm{r}}_1$ and ${\mathrm{r}}_2$ did not overlap; 50% received dose 2 uniformly between day 36 and day 42, and ${\mathrm{r}}_1$ and ${\mathrm{r}}_2$ overlapped. We then simulated cases, ${y}_t$, during ${\mathrm{c}}_0$, ${\mathrm{r}}_1$, ${\mathrm{c}}_1,{\mathrm{r}}_2$, and ${\mathrm{c}}_2$ according to the daily logit model^7^ as in previous studies^14^^,^^16^^,^^17^:


(7) \begin{equation*} \mathrm{prob}\ \left({y}_t|{k}_r\right)=\frac{\exp \left({\mathrm{\beta}}_0+{k}_1{\mathrm{\beta}}_1+{k}_2{\mathrm{\beta}}_2+{k}_{12}{\mathrm{\beta}}_{12}\right)}{1+\exp \left({\mathrm{\beta}}_0+{k}_1{\mathrm{\beta}}_1+{k}_2{\mathrm{\beta}}_2+{k}_{12}{\mathrm{\beta}}_{12}\right)} \end{equation*}

where ${y}_t$ is a binary variable for occurrence of SAEs, $\mathrm{prob}\ \left({y}_t|{k}_r\right)$ is the probability of ${y}_t$ = 1 on day $t$; $ks$ are indicators, ${k}_1=1$ if $t$ falls in the first risk interval ${\mathrm{r}}_1$, otherwise ${k}_1=0;{k}_2=1$ if $t$ falls in the second risk interval ${\mathrm{r}}_2$, otherwise ${k}_2=0$; ${k}_{12}=1$ if $t$ falls in the overlapping risk period ${\mathrm{r}}_{12}$, otherwise ${k}_{12}=0$. ${\mathrm{\beta}}_0$ determines the baseline rate of SAEs during the control intervals and equals to −9, −8, −7 in the simulation, representing annual event rates of 4.5%, 12.2%, and 33.3%, respectively. For rare SAEs, approximately, exponentiation of $\mathrm{\beta}$ is RI for a risk interval (ie, ${\mathrm{r}}_1$, ${\mathrm{r}}_2,\mathrm{and}\ {\mathrm{r}}_{12})$.

We simulated data for ${\mathrm{\beta}}_2={\mathrm{\beta}}_1$ and ${\mathrm{\beta}}_2=1.5{\mathrm{\beta}}_1$. We only described the simulation method and results for ${\mathrm{\beta}}_2={\mathrm{\beta}}_1$ due to similar results. Let ${\mathrm{\beta}}_2={\mathrm{\beta}}_1=\mathrm{\beta}$ (ie, ${\mathrm{RI}}_1={\mathrm{RI}}_2=\mathrm{RI})$, where $\mathrm{RI}=1.5,2.0,4.0$. Simulations assumed (1) a positive multiplicative effect: ${\mathrm{RI}}_{12}={\mathrm{RI}}_1{\mathrm{RI}}_2+X$, where $X$ equals 0.5, 1.0, and 2.0; (2) a positive additive and negative multiplicative effect: $\mathrm{A}<{\mathrm{RI}}_{12}<\mathrm{M}$; for simplicity, we used ${\mathrm{RI}}_{12}=\frac{\mathrm{M}+\mathrm{A}}{2}=\frac{\left({\mathrm{e}}^{2\mathrm{\beta}}+2{\mathrm{e}}^{\mathrm{\beta}}-1\right)}{2}=\frac{\left({\mathrm{RI}}^2+2\mathrm{RI}-1\right)}{2}$ because $\mathrm{A}<\frac{\left(\mathrm{A}+\mathrm{M}\right)}{2}<\mathrm{M}$. Note that because $\mathrm{M}>\mathrm{A}$, a positive multiplicative effect implies a positive additive effect.

Each of the 1000 simulated datasets was analyzed using the four methods described in the Methods section.

#### Simulation evaluation metrics

(1) Means and average SD of RIs: we reported means and SD of RIs after doses 1 and 2 from the 4 approaches and ${\mathrm{RI}}_{12}$ (SD) during the overlapping risk period from the three-part model across 1000 replicas for the 4 methods described in the Methods section. Percentage bias (%) was calculated as $\left(\frac{\mathrm{estimated}\ \mathrm{RI}-\mathrm{true}\ \mathrm{RI}}{\mathrm{true}\ \mathrm{RI}}\right)\times 100$.
(2) Percentage of correct characterization: we calculated the percentage of simulated datasets in which using RIs from the three-part model correctly characterized the risk as simulated.

All analyses were conducted using SAS Enterprise Guide 8.2 (SAS Institute Inc.). A SAS program for fitting the three-part SCCS model is available (Appendix S1). This study was approved by the KPSC Institutional Review Board.

## Results

### Simulation results

#### Mean and SD of estimated RIs

Under the assumption of a positive multiplicative effect, across different levels of RI and positive multiplicative effects during the overlapping risk period, censoring follow-up at dose 2 decreased estimation precision, indicated by increased SD for estimated ${\mathrm{RI}}_1$ and ${\mathrm{RI}}_2$ with a greater impact on ${\mathrm{RI}}_2$, while point estimates of ${\mathrm{RI}}_1$ and ${\mathrm{RI}}_2$ were largely unbiased. Including ${\mathrm{r}}_{12}$ in ${\mathrm{r}}_1$ overestimated ${\mathrm{RI}}_1$, with percentage bias ranging from 6.0% to 26.0%, and had no impact on point estimates of ${\mathrm{RI}}_2$ and SD of both ${\mathrm{RI}}_1$and ${\mathrm{RI}}_2$. Including ${\mathrm{r}}_{12}$ in ${\mathrm{r}}_2$ overestimated ${\mathrm{RI}}_2$, with percentage bias ranging from 7.3% to 34.0%, and similarly had no impact on point estimate of ${\mathrm{RI}}_1$ and SD of both ${\mathrm{RI}}_1$ and ${\mathrm{RI}}_2$ (Table 2).

**Table 2:** Under the assumption of a positive multiplicative effect^a^ during the overlapping risk period: means and SD of estimated relative incidences (RI) after doses 1 and 2 from the four methods and RI_12_ (SD) during the overlapping risk period from the three-part model.

			**Estimated parameters**								
**Simulation parameters**			**Censoring follow-up upon receiving dose 2**		**Including** ${\mathbf{r}}_{\mathbf{12}}$ **in** ${\mathbf{r}}_{\mathbf{1}}$		**Including** ${\mathbf{r}}_{\mathbf{12}}$ **in** ${\mathbf{r}}_{\mathbf{2}}$		**Three-part model**		
$\mathbf{RI}$ ^**b**^	${\mathbf{RI}}_{\mathbf{12}}$	${\boldsymbol{\mathrm{\beta}}}_{\mathbf{0}}$	${\mathbf{RI}}_{\mathbf{1}}$ **(SD)**	${\mathbf{RI}}_{\mathbf{2}}$ **(SD)**	${\mathbf{RI}}_{\mathbf{1}}$ **(SD)**	${\mathbf{RI}}_{\mathbf{2}}$ **(SD)**	${\mathbf{RI}}_{\mathbf{1}}$ **(SD)**	${\mathbf{RI}}_{\mathbf{2}}$ **(SD)**	${\mathbf{RI}}_{\mathbf{1}}$ **(SD)**	${\mathbf{RI}}_{\mathbf{2}}$ **(SD)**	${\mathbf{RI}}_{\mathbf{12}}$ **(SD)**
1.5	2.75	−9	1.55 (0.39)	1.58 (0.53)	1.62 (0.28)	1.53 (0.32)	1.53 (0.28)	1.65 (0.33)	1.52 (0.28)	1.54 (0.33)	2.85 (1.18)
		−8	1.51 (0.22)	1.53 (0.30)	1.60 (0.17)	1.51 (0.18)	1.51 (0.17)	1.63 (0.19)	1.51 (0.17)	1.52 (0.18)	2.78 (0.69)
		−7	1.50 (0.13)	1.51 (0.17)	1.59 (0.10)	1.50 (0.11)	1.50 (0.10)	1.61 (0.11)	1.50 (0.10)	1.50 (0.11)	2.75 (0.41)
2	4.5	−9	2.06 (0.48)	2.11 (0.65)	2.22 (0.36)	2.03 (0.39)	2.04 (0.34)	2.28 (0.41)	2.04 (0.34)	2.05 (0.39)	4.63 (1.52)
		−8	2.02 (0.28)	2.03 (0.35)	2.19 (0.21)	2.01 (0.22)	2.02 (0.20)	2.25 (0.23)	2.01 (0.20)	2.02 (0.22)	4.56 (0.93)
		−7	2.00 (0.16)	2.01 (0.21)	2.18 (0.13)	1.99 (0.14)	2.01 (0.12)	2.23 (0.14)	2.00 (0.12)	2.00 (0.14)	4.50 (0.54)
4	16.5	−9	4.10 (0.80)	4.19 (1.04)	4.94 (0.66)	3.99 (0.60)	4.10 (0.58)	5.22 (0.75)	4.06 (0.57)	4.08 (0.63)	16.77 (3.41)
		−8	4.01 (0.45)	4.05 (0.56)	4.89 (0.39)	3.93 (0.35)	4.06 (0.34)	5.15 (0.43)	4.02 (0.34)	4.03 (0.37)	16.56 (1.96)
		−7	4.00 (0.27)	4.01 (0.34)	4.85 (0.24)	3.91 (0.22)	4.04 (0.21)	5.10 (0.27)	4.00 (0.21)	4.00 (0.23)	16.31 (1.22)
1.5	3.25	−9	1.55 (0.39)	1.58 (0.53)	1.65 (0.29)	1.53 (0.32)	1.53 (0.28)	1.70 (0.34)	1.52 (0.28)	1.54 (0.33)	3.35 (1.29)
		−8	1.51 (0.22)	1.53 (0.30)	1.63 (0.17)	1.51 (0.18)	1.51 (0.17)	1.68 (0.19)	1.51 (0.17)	1.52 (0.18)	3.29 (0.77)
		−7	1.50 (0.13)	1.51 (0.17)	1.62 (0.10)	1.50 (0.11)	1.50 (0.10)	1.66 (0.11)	1.50 (0.10)	1.50 (0.11)	3.25 (0.45)
2	5	−9	2.06 (0.48)	2.11 (0.65)	2.25 (0.36)	2.03 (0.38)	2.04 (0.34)	2.32 (0.42)	2.04 (0.34)	2.05 (0.39)	5.15 (1.64)
		−8	2.02 (0.28)	2.03 (0.35)	2.23 (0.21)	2.00 (0.22)	2.02 (0.20)	2.29 (0.23)	2.01 (0.20)	2.02 (0.22)	5.06 (0.97)
		−7	2.00 (0.16)	2.01 (0.21)	2.21 (0.13)	1.99 (0.14)	2.01 (0.12)	2.27 (0.15)	2.00 (0.12)	2.00 (0.14)	5.00 (0.57)
4	17	−9	4.10 (0.80)	4.19 (1.04)	4.97 (0.66)	3.98 (0.60)	4.10 (0.58)	5.27 (0.75)	4.06 (0.57)	4.08 (0.63)	17.27 (3.49)
		−8	4.01 (0.45)	4.05 (0.56)	4.92 (0.39)	3.93 (0.35)	4.06 (0.34)	5.20 (0.43)	4.02 (0.34)	4.03 (0.37)	17.05 (2.01)
		−7	4.00 (0.27)	4.01 (0.34)	4.88 (0.24)	3.90 (0.22)	4.04 (0.21)	5.15 (0.27)	4.00 (0.21)	4.00 (0.23)	16.79 (1.25)
1.5	4.25	−9	1.55 (0.39)	1.58 (0.53)	1.72 (0.29)	1.52 (0.32)	1.53 (0.28)	1.79 (0.35)	1.52 (0.28)	1.54 (0.33)	4.37 (1.47)
		−8	1.51 (0.22)	1.53 (0.30)	1.70 (0.17)	1.51 (0.18)	1.52 (0.17)	1.77 (0.20)	1.51 (0.17)	1.52 (0.18)	4.31 (0.90)
		−7	1.50 (0.13)	1.51 (0.17)	1.69 (0.10)	1.49 (0.11)	1.51 (0.10)	1.75 (0.12)	1.50 (0.10)	1.50 (0.11)	4.25 (0.53)
2	6	−9	2.06 (0.48)	2.11 (0.65)	2.32 (0.37)	2.02 (0.38)	2.05 (0.34)	2.41 (0.43)	2.04 (0.34)	2.05 (0.39)	6.16 (1.82)
		−8	2.02 (0.28)	2.03 (0.35)	2.30 (0.22)	2.00 (0.22)	2.03 (0.20)	2.38 (0.24)	2.01 (0.20)	2.02 (0.22)	6.04 (1.07)
		−7	2.00 (0.16)	2.01 (0.21)	2.28 (0.13)	1.98 (0.14)	2.01 (0.12)	2.36 (0.15)	2.00 (0.12)	2.00 (0.14)	6.00 (0.63)
4	18	−9	4.10 (0.80)	4.19 (1.04)	5.04 (0.67)	3.98 (0.60)	4.10 (0.58)	5.36 (0.76)	4.06 (0.57)	4.08 (0.63)	18.28 (3.63)
		−8	4.01 (0.45)	4.05 (0.56)	4.99 (0.39)	3.92 (0.35)	4.06 (0.34)	5.29 (0.44)	4.02 (0.34)	4.03 (0.37)	18.04 (2.09)
		−7	4.00 (0.27)	4.01 (0.34)	4.95 (0.24)	3.90 (0.22)	4.04 (0.21)	5.23 (0.28)	4.00 (0.21)	4.00 (0.23)	17.77 (1.31)

a ${\mathrm{RI}}_{12}={\mathrm{RI}}_1{\mathrm{RI}}_2+\mathrm{X},$ where X is a positive number and equals 0.5, 1.0, or 2.0.

b ${\mathrm{RI}}_1={\mathrm{RI}}_2=\mathrm{RI}$ .

Under the assumption of a positive additive effect and negative multiplicative effect, similar trends were observed as under the assumption of a positive multiplicative effect, but with less magnitude. Including ${\mathrm{r}}_{12}$ in ${\mathrm{r}}_1$ overestimated ${\mathrm{RI}}_1$, with percentage bias ranging from 2.6% to 14.8%. Similarly, including ${\mathrm{r}}_{12}$ in ${\mathrm{r}}_2$ overestimated ${\mathrm{RI}}_2$, with percentage bias ranging from 4.0% to 19.3% (Table 3).

**Table 3:** Under the assumption of a positive additive and negative multiplicative effect^a^ during the overlapping risk period: means and SD of estimated relative incidences (RI) after doses 1 and 2 from the four methods and RI_12_ (SD) during the overlapping risk period from the three-part model.

			**Estimated parameters**								
**Simulation parameters**			**Censoring follow-up upon receiving dose 2**		**Including** ${\mathbf{r}}_{\mathbf{12}}$ **in** ${\mathbf{r}}_{\mathbf{1}}$		**Including** ${\mathbf{r}}_{\mathbf{12}}$ **in** ${\mathbf{r}}_{\mathbf{2}}$		**Three-part model**		
$\mathbf{RI}$ ^**b**^	${\mathbf{RI}}_{\mathbf{12}}$	${\boldsymbol{\mathrm{\beta}}}_{\mathbf{0}}$	${\mathbf{RI}}_{\mathbf{1}}$ **(SD)**	${\mathbf{RI}}_{\mathbf{2}}$ **(SD)**	${\mathbf{RI}}_{\mathbf{1}}$ **(SD)**	${\mathbf{RI}}_{\mathbf{2}}$ **(SD)**	${\mathbf{RI}}_{\mathbf{1}}$ **(SD)**	${\mathbf{RI}}_{\mathbf{2}}$ **(SD)**	${\mathbf{RI}}_{\mathbf{1}}$ **(SD)**	${\mathbf{RI}}_{\mathbf{2}}$ **(SD)**	${\mathbf{RI}}_{\mathbf{12}}$ **(SD)**
1.5	2.125	−9	1.55 (0.39)	1.58 (0.53)	1.57 (0.28)	1.53 (0.32)	1.53 (0.28)	1.60 (0.32)	1.52 (0.28)	1.54 (0.33)	2.23 (1.02)
		−8	1.51 (0.22)	1.53 (0.30)	1.55 (0.16)	1.51 (0.18)	1.51 (0.17)	1.57 (0.18)	1.51 (0.17)	1.52 (0.18)	2.15 (0.59)
		−7	1.50 (0.13)	1.51 (0.17)	1.54 (0.10)	1.50 (0.11)	1.50 (0.10)	1.56 (0.11)	1.50 (0.10)	1.50 (0.11)	2.13 (0.35)
2	3.5	−9	2.06 (0.48)	2.11 (0.65)	2.14 (0.35)	2.04 (0.39)	2.04 (0.34)	2.18 (0.40)	2.04 (0.34)	2.05 (0.39)	3.60 (1.32)
		−8	2.02 (0.28)	2.03 (0.35)	2.12 (0.21)	2.01 (0.22)	2.02 (0.20)	2.16 (0.22)	2.01 (0.20)	2.02 (0.22)	3.54 (0.80)
		−7	2.00 (0.16)	2.01 (0.21)	2.11 (0.12)	2.00 (0.14)	2.01 (0.12)	2.14 (0.14)	2.00 (0.12)	2.00 (0.14)	3.50 (0.47)
4	11.5	−9	4.10 (0.80)	4.19 (1.04)	4.59 (0.63)	4.02 (0.61)	4.08 (0.58)	4.77 (0.70)	4.06 (0.57)	4.08 (0.63)	11.77 (2.73)
		−8	4.01 (0.45)	4.05 (0.56)	4.54 (0.37)	3.97 (0.36)	4.04 (0.34)	4.70 (0.40)	4.02 (0.34)	4.03 (0.37)	11.56 (1.57)
		−7	4.00 (0.27)	4.01 (0.34)	4.51 (0.22)	3.94 (0.22)	4.02 (0.21)	4.66 (0.25)	4.00 (0.21)	4.00 (0.23)	11.40 (0.93)

a ${\mathrm{RI}}_{12}=\frac{\mathrm{A}+\mathrm{M}}{2}=\frac{\left({\mathrm{RI}}^2+2\mathrm{RI}-1\right)}{2}$ .

b ${\mathrm{RI}}_1={\mathrm{RI}}_2=\mathrm{RI}.$

Compared to the true values of RIs under which the data were simulated, the three-part model yielded unbiased estimates of ${\mathrm{RI}}_1$, ${\mathrm{RI}}_2$, and ${\mathrm{RI}}_{12}$ across different values of ${\mathrm{\beta}}_0$, RI, and $X$ under the assumption of a positive multiplicative effect (Table 2), and across different values of ${\mathrm{\beta}}_0$ and RI under the assumption of a positive additive and negative multiplicative effect (Table 3).

#### Characterizing the risk during the overlapping risk period

Under the assumption of a positive multiplicative effect, the likelihood of correctly characterizing ${\mathrm{RI}}_{12}$ increased when SAEs were common and the true risk during ${\mathrm{r}}_{12}$ was greater (ie, higher $X$ value). For example, when ${\mathrm{RI}}_1={\mathrm{RI}}_2$ = 1.5 and ${\mathrm{RI}}_{12}={\mathrm{RI}}_1{\mathrm{RI}}_2+0.5=2.75$, with ${\mathrm{\beta}}_0=-9,-8,-7$, the correct characterization percentages were 64.1%, 75.3%, and 85.6%, respectively. When ${\mathrm{RI}}_1={\mathrm{RI}}_2=1.5$ and ${\mathrm{\beta}}_0=-7$, with $X$ = 0.5, 1 and 2, the correct characterization percentages were 85.6%, 98.6%, and 100%, respectively (Table 4). Conversely, when $X$ is relatively smaller than $\mathrm{RI}$, the percentages of correct characterization decreased; for example, when $X$ = 0.5 and ${\mathrm{\beta}}_0=-7$, with $\mathrm{RI}=1.5,2.0,\mathrm{and}\ 4.0$, the percentages of correct characterization decreased from 85.6% to 58.6%.

**Table 4:** Percentage of effect types in the overlapping risk period under the assumption of a positive multiplicative effect.^a^

**Simulation parameters**			**Percentage of effect types in the overlapping risk period (%)**			
$\mathbf{RI}$ ^**b**^	${\mathbf{RI}}_{\mathbf{12}}$	${\boldsymbol{\mathrm{\beta}}}_{\mathbf{0}}$	**Positive multiplicative (correct characterization)**	**Positive additive and negative multiplicative**	**Negative additive and positive multiplicative**	**Negative additive and negative multiplicative**
1.5	2.75	−9	64.1	10.5	0.1	25.3
		−8	75.3	11.1	0	13.6
		−7	85.6	10.1	0	4.3
2.0	4.5	−9	60	25.6	0	14.4
		−8	68.5	27.4	0	4.1
		−7	77.9	21.9	0	0.2
4.0	16.5	−9	53.6	46.4	0	0
		−8	55.4	44.6	0	0
		−7	58.6	41.4	0	0
1.5	3.25	−9	77.1	8.1	0.1	14.7
		−8	89.6	7.4	0	3
		−7	98.6	1.3	0	0.1
2.0	5	−9	70.1	21.4	0	8.5
		−8	81.4	17.5	0	1.1
		−7	94.9	5.1	0	0
4.0	17	−9	58.4	41.6	0	0
		−8	63.8	36.2	0	0
		−7	72.1	27.9	0	0
1.5	4.25	−9	93	3.5	0	3.5
		−8	98.8	1.1	0	0.1
		−7	100	0	0	0
2.0	6	−9	85.1	12.7	0	2.2
		−8	95.5	4.5	0	0
		−7	99.9	0.1	0	0
4.0	18	−9	67.2	32.8	0	0
		−8	77	23	0	0
		−7	89.6	10.4	0	0

a ${\mathrm{RI}}_{12}={\mathrm{RI}}_1{\mathrm{RI}}_2+\mathrm{X},$ where X is a positive number and equals 0.5, 1.0, or 2.0.

b ${\mathrm{RI}}_1={\mathrm{RI}}_2=\mathrm{RI}$ .

Under the assumption of a positive additive and negative multiplicative effect, and given for ${\mathrm{RI}}_1={\mathrm{RI}}_2$, the percentages of correct characterization decreased with fewer SAEs (ie, smaller value for ${\mathrm{\beta}}_0$). Given a fixed value for ${\mathrm{\beta}}_0$, the percentages of correct characterization increased with higher values of ${\mathrm{RI}}_1={\mathrm{RI}}_2$ (Table 5).

**Table 5:** Percentage of effect types in the overlapping risk period under the assumption of a positive additive and negative multiplicative effect.^a^

**Simulation parameters**			**Percentage of effect types in the overlapping risk period (%)**			
$\mathbf{RI}$ ^**b**^	${\mathbf{RI}}_{\mathbf{12}}$	${\boldsymbol{\mathrm{\beta}}}_{\mathbf{0}}$	**Positive additive and negative multiplicative (correct characterization)**	**Positive additive and positive multiplicative**	**Negative additive and positive multiplicative**	**Negative additive and negative multiplicative**
1.5	2.125	−9	10.5	43.1	0.3	46.1
		−8	15.5	41.3	0	43.2
		−7	23.3	37.8	0	38.9
2.0	3.5	−9	29.5	34.8	0	35.7
		−8	43.5	28.8	0	27.7
		−7	65.9	18.8	0	15.3
4.0	11.5	−9	89.3	9.0	0	1.7
		−8	98.5	1.5	0	0
		−7	100	0	0	0

a ${\mathrm{RI}}_{12}=\frac{\mathrm{A}+\mathrm{M}}{2}=\frac{\left({\mathrm{RI}}^2+2\mathrm{RI}-1\right)}{2}$ .

b ${\mathrm{RI}}_1={\mathrm{RI}}_2=\mathrm{RI}$ .

Note that theoretically, the category of negative additive and positive multiplicative effects does not exist. However, in a simulation study, this scenario can occur when SAEs are rare. This rarity explains why there are no or very few instances of this category in Tables 4 and 5.

### Application of the three-part model to assess ischemic stroke risk following the Pfizer-BioNTech bivalent COVID-19 vaccine and influenza vaccine

In a recent SCCS study^10^ analyzing electronically identified ischemic strokes in the KPSC population, an elevated risk of ischemic stroke was found within 42 days after vaccination among individuals aged <65 years who received both the Pfizer-BioNTech bivalent COVID-19 vaccine and an influenza vaccine on the same day ($\mathrm{RI}$ = 2.13 [95% CI, 1.01-4.46]), particularly among those with a history of SARS-CoV-2 infection ($\mathrm{RI}$ = 3.94 [95% CI, 1.10-14.16]). After conducting chart review of ischemic stroke events, the RIs were no longer statistically significantly elevated. In that study, only those who received either the Pfizer-BioNTech bivalent COVID-19 vaccine alone or those who received both the Pfizer-BioNTech bivalent COVID-19 vaccine and an influenza vaccine on the same day were considered as exposed and potentially carrying an elevated risk for ischemic stroke, ignoring the potential risk after influenza vaccination that was received either alone or with the Pfizer-BioNTech bivalent COVID-19 vaccine in close proximity (not on the same day).

**Figure 3:**
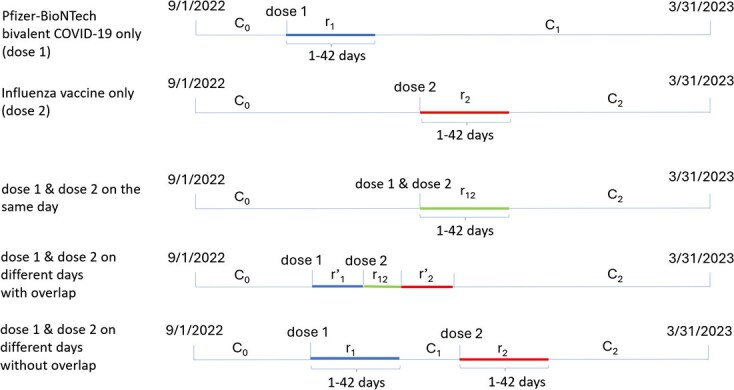
Timeline for analysis of electronically identified ischemic stroke events among members aged <65 years of Kaiser Permanente Southern California who received Pfizer-BioNTech bivalent COVID-19 and influenza vaccination during the period from September 1, 2022, to March 31, 2023.

In the current study, we re-analyzed electronically identified ischemic strokes among Pfizer-BioNTech bivalent COVID-19 and influenza vaccine recipients who were KPSC members aged <65 years from September 1, 2022, to March 31, 2023. Due to limited resources, we were unable to review and confirm the large number of electronically identified ischemic stroke events. We used the three-part model to estimate the ischemic stroke risk after the Pfizer-BioNTech bivalent COVID-19 vaccination, after influenza vaccination, and during the overlapping risk period due to coadministration of these 2 vaccines on the same day or within 42 days (as we prespecified the risk interval to be 42 days after Pfizer-BioNTech bivalent COVID-19 vaccination and after influenza vaccination). We accounted for seasonality by including the month as a covariate in the models. We assumed that the order of receiving these vaccines had no impact on the risk during the overlapping period. Additionally, we excluded day 0 (vaccination day) from the analyses, because it was indistinguishable which event occurred first, the vaccination or the ischemic stroke. The existing event-dependent SCCS cannot accommodate SAEs among 2 vaccinations administered in close proximity.^18^ To account for the impact of prior SAEs on subsequent vaccination, we excluded a 14-day washout period during the pre-vaccination interval that was not part of the overlapping risk period.^19^  Figure 3 shows the risk and control intervals for 5 hypothetical individuals who experienced ischemic strokes from September 1, 2022, to March 31, 2023.

Characteristics of those who had ischemic stroke events during the study period by type of vaccine are presented in Table S1. The number of ischemic stroke events and person-years by risk and control intervals are shown in Table S2. In the overall analysis among members aged <65 years, ischemic stroke risk was not increased after Pfizer-BioNTech bivalent COVID-19 vaccination ($\mathrm{RI}$ = 1.09 [95% CI, 0.74-1.63]) or after influenza vaccination ($\mathrm{RI}$ = 1.17 [95% CI, 0.97-1.42]) or during the overlapping risk period ($\mathrm{RI}$ = 1.20 [95% CI, 0.72-1.98]) (Table 6). For those without a history of SARS-CoV-2 infection, no vaccination was associated with an increased risk of ischemic stroke. Among individuals with a history of SARS-CoV-2 infection in the past year, the risk of ischemic stroke was increased during the overlapping risk period ($\mathrm{RI}$ = 2.74 [95% CI, 1.07-7.07]), but not after Pfizer-BioNTech bivalent COVID-19 vaccination ($\mathrm{RI}$ = 0.97 [95% CI, 0.44-2.13]) or after influenza vaccination ($\mathrm{RI}$ = 1.06 [95% CI, 0.70-1.61]). Because (${\mathrm{RI}}_{12}$ = 2.74) was greater than (${\mathrm{RI}}_1{\mathrm{RI}}_2$ = 0.97 × 1.06 = 1.03) and (${\mathrm{RI}}_1+{\mathrm{RI}}_2$ − 1 = 1.03), the risk during the overlapping risk period showed a positive multiplicative effect and a positive additive effect among those with a history of SARS-CoV-2 infection. In the overall analysis and among those without SARS-CoV-2 infection, no evidence of a positive additive or positive multiplicative effect was observed during the overlapping risk period.

**Table 6:** Relative incidences in the 42 days after Pfizer-BioNTech bivalent COVID-19 vaccination, in the 42 days after influenza vaccination, and during the overlapping risk period^a^ among members aged <65 years of Kaiser Permanente Southern California during the period from September 1, 2022 to March 31, 2023.

	**Relative incidence (95% CI)**		
	**After Pfizer-BioNTech bivalent COVID-19 vaccination**	**After influenza vaccination**	**During the overlapping risk period**
Overall	1.09 (0.74-1.63)	1.17 (0.97-1.42)	1.20 (0.72-1.98)
With history of SARS-CoV-2^b^	0.97 (0.44-2.13)	1.06 (0.70-1.61)	2.74 (1.07-7.07)
Without history of SARS-CoV-2	1.14 (0.72-1.81)	1.20 (0.96-1.50)	0.91 (0.50-1.68)

a Due to coadministration of Pfizer-BioNTech bivalent COVID-19 and influenza vaccines on the same day or in proximity.

b Had SARS-CoV-2 infection (ie, SARS-CoV-2 positive laboratory test or a COVID-19 diagnosis) during the year prior (August 31, 2021-August 31, 2022); Day 0 (vaccination day) was excluded; a 14-day washout window in control intervals before vaccinations was also excluded.

## Discussion

We proposed a three-part model for estimating three RIs separately for the overlapping risk period and after each of 2 doses when the second dose was administered before the end of the first dose’s risk interval in an SCCS design. The methods used in this study extend the prior SCCS analysis of ischemic stroke risk^10^ in several significant ways. Unlike the previous study, which did not consider the risk associated with influenza vaccination alone or the potential synergistic effects of administering COVID-19 and influenza vaccines in close proximity, this study applies a novel three-part SCCS model. This model allows for distinct characterization of risks attributable to each vaccine individually and during overlapping risk intervals, providing more nuanced insights into potential additive or multiplicative effects. By including overlapping risk periods due to coadministration on the same day or in close proximity, we address potential biases inherent in prior approaches, such as potential overestimation of risk in a single vaccine group. This approach offers a robust framework for identifying and characterizing elevated risks in overall analyses and in specific subgroups, such as individuals with a history of SARS-CoV-2 infection. Simulation studies showed that both the three-part model and censoring follow-up at ${\mathrm{d}}_2$ provided unbiased estimates for RIs while only the three-part model provided unbiased estimates for the overlapping risk period. Including the overlapping risk period in either ${\mathrm{r}}_1$ or ${\mathrm{r}}_2$ overestimated RIs for those risk intervals. Additionally, the three-part model effectively characterized risk during the overlapping period, with correct characterization rates increasing with higher baseline event rates and a greater elevated risk during the overlapping period compared to the risk after each dose alone.

We applied the three-part model to assess ischemic stroke risk after Pfizer-BioNTech bivalent COVID-19 vaccination and influenza vaccination within a 42-day risk interval among KPSC members aged <65 years from September 1, 2022, to March 31, 2023. Overall, no increased risk of ischemic stroke was found after the Pfizer-BioNTech bivalent COVID-19 vaccination or after influenza vaccination or during the overlapping risk period. Similarly, among those without a history of SARS-CoV-2 infection, no increased ischemic stroke risk was found. For those with a history of SARS-CoV-2 infection, an increased risk was observed during the overlapping risk period but not after either vaccination alone, indicating a positive multiplicative effect during the overlapping period. Chart review was not conducted to confirm ischemic stroke events when an elevated risk was detected based on electronic data due to limited resources and the large number of ischemic stroke events.

The three-part model relies on several key assumptions to estimate and characterize risk during overlapping risk periods. First, it assumes known and accurately defined risk intervals for each vaccine, which are critical for appropriately partitioning person-time into distinct risk and control intervals. Second, the model assumes that the risk due to coadministration on the same day is equivalent to the risk when vaccines are administered in close proximity, enabling a unified treatment of overlapping intervals. Third, it assumes that the order of vaccine administration does not influence the risk within the overlapping period, ensuring symmetric characterization of risk regardless of the sequence of vaccinations. Fourth, the model presumes that the risk within overlapping intervals can be independently characterized as additive, multiplicative, or both, relative to individual vaccine risks. Finally, accurate identification of SAEs is essential, as misclassification could affect risk estimates after each vaccine and after coadministration on the same day or in close proximity.^20^^,^^21^

This study has several limitations. First, in our simulation study, we did not account for situations where the SAE was negatively associated with vaccination (RI < 0). However, the study’s goal was to develop methods to assess possible elevated risks (RI > 1) after vaccination. Additionally, for simplicity, we did not include time-varying covariates (eg, seasonality) in the simulation study. However, we expect similar results if time-varying covariates were included in the simulation study. In the real-world example, we included calendar month to adjust for seasonality. Second, we assumed that the order of receiving the two vaccines did not impact the risk after each vaccination or during the overlapping risk period. Third, we did not report type I errors and empirical power in evaluating the performance of these four methods. However, an overestimated RI generally leads to inflated type I error rates under the null hypothesis and inflated empirical power under the alternative hypothesis.^16^ Fourth, the three-part model is currently designed for 2 doses, but we believe it can be extended to accommodate more doses with added complexity. Fifth, the ischemic stroke findings should be interpreted with caution because this was a single-site study with limited sample size. Additionally, ischemic stroke events were not chart reviewed, and mild transient ischemic attacks and people with a history of ischemic stroke were included.^16^ Lastly, we did not compare the three-part model to another model^12^ that parameterizes the coefficient of the overlapping risk period as a function of the risk after each vaccine and the coefficient of an interaction term.

In summary, considering the vaccination of 2 doses of the same vaccine or 1 dose each of 2 different vaccines, we propose fitting a three-part model to estimate the risk of SAEs after each vaccination and during the overlapping risk period in one SCCS analysis. By comparing the risk during the overlapping period to those after each vaccination, one can determine the characteristics of the risk during the overlapping period. A positive multiplicative effect would indicate a significant increase in risk if the 2 doses were given on the same day or in close proximity, suggesting the need for a greater interval between them. A positive additive effect but negative multiplicative effect would suggest a moderate increase in risk, suggesting that the elevated risk may be acceptable for coadministration on the same day or in close proximity, depending on the severity of the SAE. In the example, we observed that coadministration of Pfizer-BioNTech bivalent COVID-19 and influenza vaccines might be linked to an increased risk of ischemic stroke among individuals aged <65 years who had a history of COVID-19 infection in prior year. However, these findings should be interpreted cautiously due to the lack of chart review to confirm the electronically identified ischemic stroke events. Further studies with validated outcomes are needed to confirm these observations. Future research could involve applying these methods to other vaccinations to evaluate SAE risks and to validate findings across different populations and vaccination scenarios.

## Supplementary Material

Web_Material_kwaf115

## Data Availability

Individual-level data reported in this study involving human research participants are not publicly shared due to potentially identifying or sensitive patient information. Upon request to the corresponding author, and subject to review and approval of an analysis proposal, KPSC may provide the deidentified aggregate-level data that support the findings of this study within 6 months. Anonymized data (deidentified data including participant data as applicable) that support the findings of this study may be made available from the investigative team in the following conditions: (1) agreement to collaborate with the study team on all publications, (2) provision of external funding for administrative and investigator time necessary for this collaboration, (3) demonstration that the external investigative team is qualified and has documented evidence of training for human subjects protections, and (4) agreement to abide by the terms outlined in data use agreements between institutions.

## References

[1] France EK, Glanz J, Xu S, et al. Risk of immune thrombocytopenic purpura after measles-mumps-rubella immunization in children. *Pediatrics*. 2008;121(3):e687-e692. 10.1542/peds.2007-1578 18310189

[2] O’Leary ST, Glanz JM, McClure DL, et al. The risk of immune thrombocytopenic purpura after vaccination in children and adolescents. *Pediatrics*. 2012;129(2):248-255. 10.1542/peds.2011-1111 22232308

[3] Greene SK, Rett M, Weintraub ES, et al. Risk of confirmed Guillain-Barre syndrome following receipt of monovalent inactivated influenza a (H1N1) and seasonal influenza vaccines in the vaccine safety datalink project, 2009-2010. *Am J Epidemiol*. 2012;175(11):1100-1109. 10.1093/aje/kws195 22582210 PMC6272801

[4] CDC . Interim Clinical Considerations for Use of COVID-19 Vaccines Currently Approved or Authorized in the United States. Published June 2, 2021. Accessed on April 22, 2024. https://www.cdc.gov/vaccines/covid-19/clinical-considerations/covid-19-vaccines-us.html

[5] Grohskopf LA, Blanton LH, Ferdinands JM, et al. Prevention and control of seasonal influenza with vaccines: recommendations of the advisory committee on immunization practices - United States, 2022-23 influenza season. *MMWR Recomm Rep*. 2022;71(1):1-28. 10.15585/mmwr.rr7101a1 PMC9429824 36006864

[6] Izikson R, Brune D, Bolduc JS, et al. Safety and immunogenicity of a high-dose quadrivalent influenza vaccine administered concomitantly with a third dose of the mRNA-1273 SARS-CoV-2 vaccine in adults aged ≥65 years: a phase 2, randomised, open-label study. *Lancet Respir Med*. 2022;10(4):392-402. 10.1016/S2213-2600(21)00557-9 35114141 PMC8803382

[7] Gonen T, Barda N, Asraf K, et al. Immunogenicity and Reactogenicity of Coadministration of COVID-19 and influenza vaccines. *JAMA Netw Open*. 2023;6(9):e2332813. 10.1001/jamanetworkopen.2023.32813 37682571 PMC10492184

[8] Andrews N, Stowe J, Miller E, et al. BA.1 bivalent COVID-19 vaccine use and stroke in England. *JAMA*. 2023;330(2):184-185. 10.1001/jama.2023.10123 37318811 PMC10273126

[9] Lu Y, Matuska K, Nadimpalli G, et al. Stroke risk after COVID-19 bivalent vaccination among US older adults. *JAMA*. 2024;331(11):938-950. 10.1001/jama.2024.1059 38502075 PMC10951737

[10] Xu S, Sy LS, Hong V, et al. Ischemic stroke after bivalent COVID-19 vaccination: self-controlled case series study. *JMIR Public Health Surveill*. 2024;10:e53807-e53807. 10.2196/53807 38916940 PMC11234065

[11] Escolano S, Hill C, Tubert-Bitter P. A new self-controlled case series method for analyzing spontaneous reports of adverse events after vaccination. *Am J Epidemiol*. 2013;178(9):1496-1504. 10.1093/aje/kwt128 24013203

[12] Whitaker HJ, Paddy Farrington C, Spiessens B, et al. Tutorial in biostatistics: the self-controlled case series method. *Stat Med*. 2006;25(10):1768-1797. 10.1002/sim.2302 16220518

[13] Farrington CP . Relative incidence estimation from case series for vaccine safety evaluation. *Biometrics*. 1995;51(1):228-235. 10.2307/2533328 7766778

[14] Xu S, Zeng C, Newcomer S, et al. Use of fixed effects models to analyze self-controlled case series data in vaccine safety studies. *J Biom Biostat*. 2012;1(S7). 10.4172/2155-6180.s7-006 PMC3976179 24707443

[15] VanderWeele TJ, Knol MJ. A tutorial on interaction. *Epidemiol Methods*. 2014;3(1):33-72. 10.1515/em-2013-0005

[16] Xu S, Sy LS, Han B, et al. A propensity score approach and a partitioned approach for the self-controlled case series design to evaluate safety of a 2-dose vaccine series: application to myocarditis/pericarditis following mRNA COVID-19 vaccination. *Am J Epidemiol*. 2024;194(1):254-266. 10.1093/aje/kwae141 PMC11735979 38907283

[17] Xu S, Hambidge SJ, McClure DL, et al. A scan statistic for identifying optimal risk windows in vaccine safety studies using self-controlled case series design. *Stat Med*. 2013;32(19):3290-3299. 10.1002/sim.5733 23303643 PMC4004030

[18] Farrington P, Whitaker H, Ghebremichael-Weldeselassie Y. Self-Controlled Event Series Studies: A Modelling Guide With R. Boca Raton: FL. Chapman & Hall/CRC Press; 2018.

[19] Lee JJY, Bernatsky S, Benchimol EI, et al. COVID-19 vaccination safety and associated health care utilization among adults with inflammatory bowel disease - a population-based self-controlled case series analysis. *BMC Gastroenterol*. 2024;24(1):189. 10.1186/s12876-024-03273-0 38816836 PMC11137996

[20] Xu S, Newcomer S, Nelson J, et al. Signal detection of adverse events with imperfect confirmation rates in vaccine safety studies using self-controlled case series design. *Biom J*. 2014;56(3):513-525. 10.1002/bimj.201300012 24402780

[21] Newcomer SR, Kulldorff M, Xu S, et al. Bias from outcome misclassification in immunization schedule safety research. *Pharmacoepidemiol Drug Saf*. 2018;27(2):221-228. 10.1002/pds.4374 29292551 PMC5800415

