# Supplementary material for: A three-part model for the self-controlled case series design to estimate and characterize adverse event risk in an overlapping risk period after multiple vaccines: application to ischemic stroke following Pfizer-BioNTech bivalent COVID-19 vaccine and influenza vaccine
Source: Am J Epidemiol. 2025 Jun 4;195(4):956–65. doi: 10.1093/aje/kwaf115 (PMC13066344; doi:10.1093/aje/kwaf115)
Supplement: Web_Material_kwaf115 [file web_material_kwaf115.docx]

**A Three-Part Model for the Self-Controlled Case Series Design to** **Estimate and Characterize Adverse Event Risk in an Overlapping Risk Period after Multiple Vaccines: Application to Ischemic Stroke Following Pfizer-BioNTech Bivalent COVID-19 Vaccine and Influenza Vaccine**

Stanley Xu, PhD^1,2^, Lina S. Sy, MPH^1^, Xuan Huang, MS^1^, Vennis Hong, MPH^1^, Bing Han, PhD^1^, Katia J. Bruxvoort, PhD^1,3^, Bruno Lewin MD^1,4^, Kimberly J. Holmquist, MPH^1^, Lei Qian, PhD^1^

^1^Department of Research & Evaluation, Kaiser Permanente Southern California, Pasadena, CA

^2^Department of Health Systems Science, Kaiser Permanente Bernard J. Tyson School of Medicine, Pasadena, CA

^3^School of Public Health, University of Alabama at Birmingham, Birmingham, AL

^4^Department of Clinical Science, Kaiser Permanente Bernard J. Tyson School of Medicine, Pasadena, CA

**Supplementary Materials**

APPENDIX S1. SAS codes for fitting the three-part SCCS model

Table S1. Characteristics of individuals who had ischemic stroke events by type of vaccine received among members of Kaiser Permanente Southern California aged <65 years old from September 1, 2022, to March 31, 2023

Table S2. Number of electronically identified ischemic stroke events and person-years by risk (1-42 day after Pfizer-BioNTech bivalent COVID-19 and influenza vaccination) and control intervals§ among members aged <65 years of Kaiser Permanente Southern California during the period from September 1, 2022, to March 31, 2023.

APPENDICES.

APPENDIX S1. SAS codes for fitting the three-part SCCS model

/****************************************************************************

* Title: SAS codes for fitting the three-part SCCS model after 2 vaccines

* Developer: Stanley Xu, PhD

* Research and Evaluation

* Kaiser Permanente Southern California

* Email: stan.xu@kp.org

* Date Created: 07/28/2024

****************************************************************************/

libname mydir 'S:\R&E Scientists\Stanley Xu\';

/********************************************************************************************************;

*The SCCS dataset, mydir.pfizer_influenza_cases, has ischemic outcome events during the study period, 9/1/2022-3/31/2023, among those aged <65 years. The dataset has the following variables:

*case, a unique number for each case

*age, age on 9/1/2022

*bivalent1, the date when the 1st dose bivalent covid-19 vaccine was given

*bivalent2, the date when the 2nd dose bivalent covid-19 vaccine was given

*the maximum number of influenza vaccine doses was 3

*influenza1, the date when the 1st dose influenza vaccine was given

*influenza2, the date when the 2nd dose influenza vaccine was given

*influenza3, the date when the 3rd dose influenza vaccine was given

*admitdate, date when an ischemic stroke event occurred

*covid_prior, indicator for covid-19 infection within 1 year prior to 9/1/2022

*death, an indicator if one died during the study period

*death_date, date of death

*******************************************************************************************************/

/***************************************************************************;

*Step 1, prepare data for the three-part model

*create new variables for those dates anchored on 9/1/2022

*fup was censored at death, disenrollment, receiving the 2nd dose of covid-19 vaccine, or end *of the study period, which ever came first

*vgrp, a variable for groups who received only influenza vaccine, only covid-19 vaccine, both on *the day same day, and both on different day

****************************************************************************/

**data** pfizer_influenza_cases1;

length vgrp $ **15**;

set mydir.pfizer_influenza_cases;

fup_start=mdy(**9**,**1**,**2022**);

fup_end=min(mdy(**3**,**31**,**2023**), death_date, disenroll,bivalent2);

covid_vday=bivalent1-mdy(**9**,**1**,**2022**)+**1**;

influenza_vday1=influenza1-mdy(**9**,**1**,**2022**)+**1**;

influenza_vday2=influenza2-mdy(**9**,**1**,**2022**)+**1**;

influenza_vday3=influenza3-mdy(**9**,**1**,**2022**)+**1**;

event_day=admitdate-mdy(**9**,**1**,**2022**)+**1**;

fu_start_day=**1**;

fu_end_day=fup_end-mdy(**9**,**1**,**2022**)+**1**;

if covid_vday=**.** and influenza_vday1=**.** then vgrp='unexp';

else if covid_vday=**.** and influenza_vday1 ne **.** then vgrp='flu';

else if covid_vday ne **.** and influenza_vday1=**.** then vgrp='covid';

else if covid_vday ne **.** and ((influenza_vday1 ne **.** and covid_vday=influenza_vday1)

or (influenza_vday2 ne **.** and covid_vday=influenza_vday2)

or (influenza_vday3 ne **.** and covid_vday=influenza_vday3)) then vgrp='same day';

else if covid_vday ne **.** and (influenza_vday1 ne **.** or influenza_vday2 ne **.** or influenza_vday3 ne **.**) then vgrp='diff day';

**run**;

option nocenter;

**proc** **freq** data=pfizer_influenza;

tables vgrp;

**run**;

*Step 2: expand the data into daily observation with indicators for exposures and outcomes;

*Analyzing SCCS data using the approach in Xu et al Journal of Data Science 8(2010), 349-360;

*Step 2a: pre-specify risk intervals after covid and flu vaccinations;

%let covid_risk_int_len=42;

%let influenza_risk_int_len=42;

*Step 2b: expand the data into daily observation;

**data** pfizer_influenza_day;

set pfizer_influenza;;

do day=fu_start_day to fu_end_day;

surv=**1**; **for count person time;

*create the seasonality variable 7 months;

if day<=**30** then season=**1**;

else if day<=**61** then season=**2**;

else if day<=**91** then season=**3**;

else if day<=**122** then season=**4**;

else if day<=**150** then season=**5**;

else if day<=**181** then season=**6**;

else season=**7**;

*create an indicator for outcome event on each day;

if day=event_day then outcome_event=**1**;

else outcome_event=**0**;

*create an indicator for risk and control intervals for COVID-19 vaccination;

if covid_vday=<day=<(covid_vday+&covid_risk_int_len-**1**) then do;

covid_risk_int=**1**;

end;

else covid_risk_int=**0**;

*create indicators for risk and control intervals for influenza vaccination;

if influenza_vday1=<day=<(influenza_vday1+&influenza_risk_int_len-**1**) then influenza_risk_int=**1**;

else if influenza_vday2=<day=<(influenza_vday2+&influenza_risk_int_len-**1**) then influenza_risk_int=**1**;

else if influenza_vday3=<day=<(influenza_vday3+&influenza_risk_int_len-**1**) then influenza_risk_int=**1**;

else influenza_risk_int=**0**;

output;

end;

**run**;

*Step 3: define the type of the risk intervals and control interval for each day;

**data** pfizer_influenza_day1;

length grp $ **15**;

set pfizer_influenza_day;

if covid_risk_int=**0** and influenza_risk_int=**0** then grp='control'; ***controls;

else if covid_risk_int=**1** and influenza_risk_int=**0** then grp='covid risk';

else if covid_risk_int=**0** and influenza_risk_int=**1** then grp='flu risk';

else if covid_risk_int=**1** and influenza_risk_int=**1** then grp='overlap risk';

**run**;

*Step 4: 1) remove day 0, 2) exclude pre-specified washout window (WOW) in control intervals preceding vaccinations;

* or comment out the relevant Syntax without considering WOW;

%let wow=your wow;

**data** pfizer_influenza_day2;

set pfizer_influenza_day1;

*remove day 0 from risk interval for influenza and covid vaccines;

if day-influenza_vday1=**0** or day-influenza_vday2=**0** or day-influenza_vday3=**0** or day-Covid_vday=**0** then delete;

*if grp='control' and (-&wow<=day-influenza_vday1<0 or -&wow<=day-influenza_vday2<0 or -&wow<=day-influenza_vday3<0 or -&wow<=day-covid_vday<0) then delete;

**run**;

*Step 5: to estimate relative incidences after each vaccination and during the overlapping risk period;

*Step 5a: overall analyses among those<65 years old;

**PROC** **PHREG** DATA=pfizer_influenza_day2;

class grp(ref='control') season;

MODEL surv * outcome_event (**0**) = grp season /TIES=BRESLOW RISKLIMITS ALPHA=**0.05**;

ods output parameterestimates=overall_under65;

strata case;

**run**;

*Step 5b: among those <65 and who had a history of COVID-19 infection in 1 year prior to 9/1/2022;

**PROC** **PHREG** DATA=pfizer_influenza_day2;

where covid_prior=**1**;

class grp(ref='control') season;

MODEL surv * outcome_event (**0**) = grp season /TIES=BRESLOW RISKLIMITS ALPHA=**0.05**;

ods output parameterestimates=overall_under65_covid_prior;

strata case;

**run**;

*Step 5c: among those aged<65 and who did not have a history of COVID-19 infection in 1 year prior to 9/1/2022;

**PROC** **PHREG** DATA=pfizer_influenza_day2;

where covid_prior=**0**;

class grp(ref='control') season;

MODEL surv * outcome_event (**0**) = grp season /TIES=BRESLOW RISKLIMITS ALPHA=**0.05**;

ods output parameterestimates=overall_under65_nocovid_prior;

strata case;

**run**;

**Table S1**. Characteristics of individuals who had ischemic stroke events by type of vaccine received among members of Kaiser Permanente Southern California aged <65 years old from September 1, 2022, to March 31, 2023

|  | Only Pfizer-BioNTech bivalent COVID-19 vaccination | Only influenza vaccination | Both on the same day | Both on different days | Did not receive either | Entire sample |
| --- | --- | --- | --- | --- | --- | --- |
| **Sample size** | 43 | 533 | 36 | 142 | 530 | 1,284 |
| **mean age (years)** (std) | 53.8 (8.0) | 52.8 (9.4) | 54.4 (9.4) | 54.1 (8.9) | 50.7 (10.3) | 52.2 (9.8) |
| **Female**, n (%) | 24 (55.8) | 281 (52.7) | 16 (44.4) | 59 (41.5) | 259 (48.9) | 639 (49.8) |
| **Race ethnicity** |  |  |  |  |  |  |
| Asian/Pacific Islander | 5 (11.6) | 61 (11.4) | 3 (8.3) | 17 (12.0) | 35 (6.6) | 121 (9.4) |
| Black | 6 (14.0) | 67 (12.6) | 7 (19.4) | 9 (6.3) | 78 (14.7) | 167 (13.0) |
| Hispanic | 17 (39.5) | 263 (49.3) | 15 (41.7) | 66 (46.5) | 247 (46.6) | 608 (47.4) |
| White | 14 (32.6) | 127 (23.8) | 11 (30.6) | 48 (33.8) | 145 (27.4) | 345 (26.9) |
| Other/Unknown | 1 (2.3) | 15 (2.8) | 0 (0.0) | 2 (1.4) | 25 (4.7) | 43 (3.4) |

**Table S2.** Number of electronically identified ischemic stroke events and person-years by risk (1-42 day after Pfizer-BioNTech bivalent COVID-19 and influenza vaccination) and control intervals^§^ among members aged <65 years of Kaiser Permanente Southern California during the period from September 1, 2022, to March 31, 2023.

| **Risk and control intervals** | Number of ischemic stroke events | Person-years |
| --- | --- | --- |
| **Risk interval after Pfizer-BioNTech bivalent COVID-19 vaccination** | 32 | 20 |
| **Risk interval after influenza vaccination** | 157 | 100 |
| **Overlapping risk interval** | 20 | 10 |
| **Control interval** | 1075 | 680 |
| **Total** | 1,284 | 810 |

^§^ Individuals vaccinated with either or both vaccines contributed to both the risk and control intervals, while those who were not vaccinated with either vaccine contributed only to the control intervals.
